# Multi-omics and perturbation screens as discovery tools in immunometabolism

**DOI:** 10.1371/journal.pbio.3003570

**Published:** 2026-01-09

**Authors:** Nicole M. Chapman, Hongbo Chi

**Affiliations:** Department of Immunology, St Jude Children’s Research Hospital, Memphis, Tennessee, United States of America; University of Birmingham, UNITED KINGDOM OF GREAT BRITAIN AND NORTHERN IRELAND

## Abstract

Metabolic regulation of immune function is a complex and dynamic process. This Perspective highlights how systems biology approaches, including multi-omics profiling and perturbomics screening, are being used to unravel this complexity and advance understanding of immunometabolism.

## Teaser

Metabolic rewiring of immune cells has broad impacts on immune responses and disease outcomes. Systems biology approaches, such as multi-omics profiling and perturbation screening, could uncover new actionable targets and therapeutic avenues to explore.

Systemic and cellular metabolic processes are critical regulators of immune cell function. Indeed, extracellular nutrients, metabolic rewiring, and metabolism-associated signaling mediators orchestrate immune cell fate, state, and functional outcomes [1]. Initial studies in the field of immunometabolism often applied “reductionist” approaches (e.g., analysis of cell type-specific gene knockout mice), which are powerful for establishing causality in metabolic effects and dependency. However, they do not fully recapitulate the complex intracellular metabolic pathways or the interplay that occurs between immune cells and their microenvironment in vivo. As a result, we still lack a systems-level understanding of the metabolic processes underpinning the functional adaptation of immune cells in diverse contexts, especially at the single-cell or spatial level in vivo.

**Fig 1:**
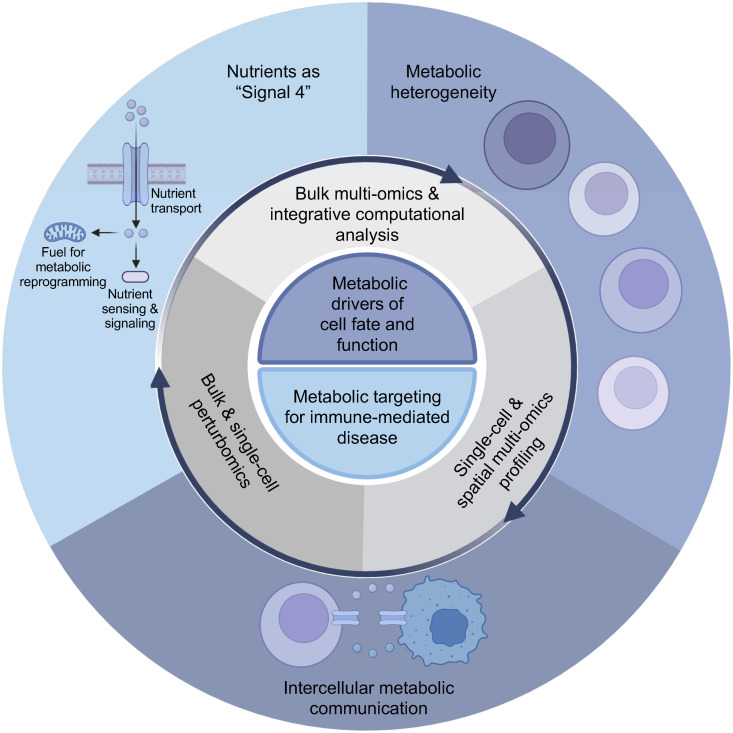
Systems biology approaches to investigate emerging concepts in immunometabolism. The figure illustrates three emerging concepts in immunometabolism research: nutrients as “Signal 4” (which includes effects on metabolic rewiring and/or nutrient/metabolite-dependent signaling), metabolic heterogeneity (i.e., cells with different metabolic states such as preferential use of glycolysis or oxidative phosphorylation, represented by different colored and sized cells) and intercellular metabolic communication (outer circle). These concepts will be greatly advanced by using integrative approaches to combine functional perturbation analyses using CRISPR-based platforms with multi-omics approaches to profile the transcriptional, epigenetic, and metabolic states of immune cells, including at the bulk, single-cell, and spatial levels (middle circle). Ultimately, the fundamental discoveries made with these integrative systems biology tools will advance our understanding of immunology and the application of targeting metabolism-associated processes to treat immune-mediated diseases (inner circle). Figure created in BioRender. Chapman, N. (2025) https://BioRender.com/vyudh54.

Systems biology approaches have transformed our understanding of immunity. In particular, single-cell transcriptome profiling has revealed molecular pathways and heterogeneous cell states that correlate with disease progression or therapeutic outcomes, with metabolome, epigenome, and proteome analyses providing further insights. Nonetheless, these profiling approaches alone often fail to uncover the causal drivers underlying cell fate and function. Integrative applications of genetic perturbation approaches (especially CRISPR–Cas9-based genetic screens), multi-omics profiling, and computational biology approaches have therefore been adopted to uncover such functional drivers [2]. These systems-level multi-omics and “perturbomics” approaches have led to fundamental discoveries and mechanisms in immunology that cannot be surmised from simpler systems, thereby uncovering new biological insights and actionable targets for cancer and other diseases. Integrating such systems biology tools will be vital to help researchers address emerging concepts in immunometabolism, such as the role of nutrients as “Signal 4,” metabolic heterogeneity, and intercellular metabolic communication (Fig 1). Here, we discuss the application of systems biology tools, including bulk, single-cell, and spatial platforms, to address these emerging concepts, using T cells as an example.

Recently, the concept that extracellular nutrients can serve as “Signal 4” in T cell biology has emerged [3]. T cells must continuously sense and adapt to changes in their environmental milieu. Three major classes of immune signals (cognate antigen, co-stimulation, and cytokines) have long been known to orchestrate the activation and functional adaptation of T cells, and these signals also influence metabolic reprogramming in T cell biology [1]. Because nutrient transporters acquire extracellular nutrients that function as both metabolic fuel sources and signaling molecules, extracellular nutrients have recently emerged as a fourth class of signals to instruct T cell responses. However, nutrient composition differs across and within tissues and is subject to undergo further alterations in the context of disease, complicating our understanding of how nutrients shape immune responses.

A multi-pronged approach will be necessary to understand how local nutrient availability affects T cell responses. First, an increasing number of studies highlight that T cells metabolically adapt to alterations in nutrient composition, which can be revealed using a combination of approaches, including in vivo stable isotope tracing studies and tissue-level metabolomics profiling, together with individual and combinatorial targeting of metabolic pathways in vivo. Second, it will be essential to uncover the requirement of T cells for different nutrients in diverse contexts. This concept could be explored via modulation of nutrients (for instance, manipulating nutrient composition in the diet or directly in the microenvironment) and their transporters (for example, genetic or pharmacological perturbations) [3]. Third, although much focus has been placed on the metabolic role of nutrients as fuel sources, less is understood about their signaling effects, especially beyond epigenetic and transcriptional regulation. As nutrient-dependent signaling events further contribute to metabolic rewiring and vice versa [3], it will also be essential to uncover the intracellular sensors and signal transducers, enabling discovery of new immunometabolic mechanisms. To this end, a recent genome-wide CRISPR screen identified regulators of nutrient-sensitive mTORC1 signaling. Overlapping the “hits” from this screen with protein–protein interaction network analysis revealed key signaling nodes underlying three-tiered nutrient signaling, composed of nutrient transporters, sensors, and transducers, and their functional effects on shaping T cell biology [4]. These approaches, together with emerging techniques to profile metabolite–RNA or metabolite–protein interactions, will greatly advance our mechanistic understanding of nutrient signaling and “Signal 4” in adaptive immunity.

The concept of metabolic heterogeneity (or heterogenous metabolic states) underlying T cell function has also recently been introduced [1,5]. During immune responses, discrete functional subsets of T cells, such as diverse CD4^+^ T helper cell populations, are generated, and metabolic reprogramming contributes to their differentiation and functional specialization [1]. Furthermore, T cell transitional states with discrete metabolic regulation or functional effects in adaptive immune and therapeutic responses often emerge. The identification of these metabolically heterogenous cell subsets or transitional states has been accelerated by the applications of single-cell metabolic profiling techniques. These include flow cytometry-based assays, such as SCENITH or MetFlow, and computational algorithms like COMPASS, which can be used to infer metabolic heterogeneity from single-cell transcriptomics data [6]. Moreover, by integrating these platforms with computational algorithms (e.g., NetBID) and genetic perturbation studies, metabolism-associated “hidden drivers” (functional molecules not regulated at the gene or protein level) underlying metabolic heterogeneity can be uncovered. Indeed, metabolism-associated enzymes or regulators are often affected by post-transcriptional or post-translational mechanisms and therefore may not be captured in conventional differential expression analyses.

Bulk CRISPR screens combined with metabolic profiling have been foundational in uncovering metabolic regulators of T cell fate decisions [2]. However, heterogenous metabolic states often represent rare or transient populations, limiting our ability to perform conventional metabolic profiling assays. Furthermore, identifying markers that enable screening for factors driving metabolic heterogeneity of transitional states, such as through bulk CRISPR screens, remains challenging. To address these gaps, we propose the application of cutting-edge, single-cell technologies, especially single-cell CRISPR screening platforms [7] and single-cell metabolic profiling approaches [8]. These integrated methodologies are expected to uncover the metabolic regulatory networks underlying the generation of heterogenous states, thereby accelerating how metabolic heterogeneity can be targeted to treat conditions such as cancer and autoimmune diseases.

A third emerging concept is how nutrients and metabolites influence the cell–cell communication that regulates the initiation and outcomes of immune responses. To understand this intercellular metabolic communication, seminal studies have profiled the composition of nutrients and metabolites in discrete tissue microenvironments (such as the tumor microenvironment or inflamed tissues) and combined this profiling with experiments to identify the cellular source(s) of these nutrients/metabolites and their functional effects on specific immune cell populations. This information, together with targeted deletion of intracellular metabolic enzymes or nutrient transporters in cell types of interest, has led to fundamental discoveries in intercellular metabolic communication and its impact on disease and therapies. Nonetheless, it remains challenging to understand how intercellular metabolic communication within complex tissue microenvironments shapes immune outcomes in vivo. Emerging in vivo spatial CRISPR-based screening technologies [2], especially those combining in situ detection of perturbations (e.g., guide RNA detection) with spatial transcriptomics [9] (including whole RNA transcriptomes beyond protein-coding RNAs [10]), proteomics, or metabolomics, will likely uncover mechanisms of spatially resolved intercellular communication, including the effects of metabolic factors.

We envision that the next era of immunometabolic research will benefit from the integrative applications of high-dimensional perturbation screens, multi-omics profiling, and computational biology approaches. In particular, high-dimensional, single-cell or spatial perturbomics, combined with the use of artificial intelligence and machine learning models, will allow for the reconstruction of causal gene and metabolic regulatory networks, ushering in an era of causal systems biology in immunometabolism. We anticipate that such investigations will provide new and fundamental biological insights into immunometabolism and transform treatments for immune-mediated diseases.
